# A Novel Dual-Targeted α-Helical Peptide With Potent Antifungal Activity Against Fluconazole-Resistant *Candida albicans* Clinical Isolates

**DOI:** 10.3389/fmicb.2020.548620

**Published:** 2020-09-30

**Authors:** Yang Yang, Chenxi Wang, Nan Gao, Yinfeng Lyu, Licong Zhang, Sujiang Zhang, Jiajun Wang, Anshan Shan

**Affiliations:** ^1^Laboratory of Molecular Nutrition and Immunity, Institute of Animal Nutrition, Northeast Agricultural University, Harbin, China; ^2^Key Laboratory of Tarim Animal Husbandry Science and Technology, College of Animal Science, Tarim University, Alar, China

**Keywords:** antimicrobial peptides, antifungal activity, fluconazole resistance, *Candida albicans*, membrane disruption, ROS production

## Abstract

Due to compromised immune system, fungal infection incidences have markedly increased in the last few decades. Pathogenic fungi have developed resistance to the clinically available antifungal agents. Antifungal resistance poses a great challenge to clinical treatment and has stimulated the demand for novel antifungal agents. A promising alternative to the treatment of fungal diseases is the use of antimicrobial peptides (AMPs). However, the antifungal activities of AMPs have not been fully determined. Therefore, this study aimed at designing and screening α-helical peptides with potential antifungal activities. The effects of key physicochemical parameters on antifungal activities were also investigated. A series of lengthened and residue-substituted derivatives of the template peptide KV, a hexapeptide truncated from the α-helical region of porcine myeloid antimicrobial peptide-36, were designed and synthesized. Enhancement of hydrophobicity by introducing aromatic hydrophobic amino acids (tryptophan and phenylalanine) significantly increased the efficacies of the peptides against *Candida albicans* strains, including fluconazole-resistant isolates. Increased hydrophobicity also elevated the toxic properties of these peptides. RF3 with moderate hydrophobicity exhibited potent anticandidal activities (GM = 6.96 μM) and modest hemolytic activities (HC10 > 64 μM). Additionally, repeated exposure to a subinhibitory concentration of RF3 did not induce resistance development. The antifungal mechanisms of RF3 were due to membrane disruptions and induction of reactive oxygen species production. Such a dual-targeted mechanism was active against drug-resistant fungi. These results show the important role of hydrophobicity and provide new insights into designing and developing antifungal peptides. Meanwhile, the successful design of RF3 highlights the potential utility of AMPs in preventing the spread of drug-resistant fungal infections.

## Introduction

Antimicrobial resistance (AMR) is a serious public health concern globally. The burden and consequences of antifungal resistance have not been fully recognized (Sanglard, 2016; Fisher et al., 2018). In the past few decades, incidences of fungal infections have increased dramatically due to impaired immune systems, complex surgical procedures, and extensive antibiotic treatment (Andes et al., 2016; Limper et al., 2017; Vazquez, 2017). The *Candida* species of fungi are frequently encountered as etiological agents of nosocomial infections. Infections due to *Candida* range from cutaneous to severe invasive infections (Pappas et al., 2004; Fesharaki et al., 2013). The *Candida albicans* (*C. albicans*) strain of this species is the most prevalent and is responsible for more than 50% of human candidiasis cases (Zhang et al., 2015). Despite this prevalence, therapeutic options are limited. Currently, there are three classes of antifungal drugs used to treat severe *C. albicans* infections. They include the azoles, the echinocandins, and the polyenes (Odds et al., 2003; Nett and Andes, 2016). Resistance to first- and second-line antifungals, such as echinocandins and fluconazole (FLU), are a major challenge toward *C. albicans* infections (Whaley et al., 2016; Bhetariya et al., 2017). Resistance has necessitated efforts toward the development of novel antifungal agents.

The potent, broad-spectrum endogenous antibiotics, antimicrobial peptides (AMPs), have emerged as a rising star in the field of drug research. As an intrinsic part of the innate immune system, AMPs exist widely in nearly all living organisms, providing the first line of defense against a broad range of invading microorganisms (Broekaert et al., 1995; Vos et al., 1995; Ganz and Lehrer, 1998). AMPs have diverse structures, biological functions, and molecular targets. Therefore, a systematic classification of AMPs will improve the efficiency in AMP research. AMPs can be classified into four groups based on their secondary structures, namely, liner α-helical peptides, β-sheet-containing peptides, peptides involving α- and β-elements, and extended peptides (Koehbach and Craik, 2019). The α-helical peptides are the most frequently encountered in nature and have been extensively studied due to their high synthetic accessibility. Alpha-helical peptides share common characteristics that contribute to their biological activities. These characteristics include positive charges, hydrophobicity, and amphiphilicity that disrupt microbial membranes. Upon electrostatic interactions with negatively charged cell membranes, these peptides assume secondary conformations that facilitate the insertion of their hydrophobic regions into the cell membrane (Nguyen et al., 2011; Wang et al., 2019). Moreover, AMPs can also be classified based on their antimicrobial activities. These functions could be antibacterial, antifungal, antiviral, and antiparasitic (Lacerda et al., 2016; Kodedová and Sychrová, 2017; Methatham et al., 2017; Chou et al., 2019). In addition to the ability to fight against microbes, some AMPs exhibit antioxidant, anti-inflammatory, and wound healing properties (Harioudh et al., 2017; Williams et al., 2018; Dou et al., 2019). Studies aimed at improving the antibacterial activities of AMPs and confirming the efficacy of antibacterial peptides designed by sequence modifications from naturally occurring, templated peptides or *de novo* design of a few amino acids have been done (Shao et al., 2018; Yang et al., 2019, 2020). However, the antifungal activities of the AMPs have only been partially explored. In addition to the earlier mentioned methods of classification, AMPs can be classified into cell membrane- and intracellular-targeting peptides (Lécorché et al., 2012; Li et al., 2017). Three membrane-disrupting mechanisms of AMPs have been proposed. They include barrel-stave, carpet, and toroidal mechanisms (Li et al., 2017). Additionally, many AMPs exhibit antimicrobial effects by interacting with intracellular target molecules (e.g., proteins, DNA, and RNA) or by acting as metabolic inhibitors (Lupetti et al., 2002; Le et al., 2017). Such multifaceted mechanisms of action reduce the development of microbial drug resistance.

After considering the secondary structure (membrane-disrupting property of α-helix), biological functions (not fully explored antifungal activity), and mechanism of action (decreased probability of resistance due to a multifaceted mechanism of action), we designed and screened α-helical peptides that target *C. albicans* with multiple mechanisms. The different physicochemical and structural properties of the antifungal activities of AMPs were investigated. From a hexapeptide named KV, truncated from the α-helical region of porcine myeloid antimicrobial peptide-36 (Lv et al., 2014), a series of peptides were designed. To obtain proper amphiphilicity, the reverse tandem duplication method was used to increase the length of the peptides (designated as KV2 and KV3). An arginine (Arg)-substituted analog was then designed by replacing the lysine (Lys) with Arg to increase its cationic ability (designated as RV3). Finally, valine (Val) residues were replaced with aromatic hydrophobic amino acids. Tryptophan (Trp) and phenylalanine (Phe) were replaced separately on the lysine-containing peptide (KV3) and arginine-containing peptide (RV3) to obtain KF3, KW3, RF3, and RW3.

The secondary structures of the designed peptides were determined using circular dichroism (CD) spectroscopy in aqueous and membrane-mimetic media. Evaluation of the antifungal efficacy of these peptides was done by determining their minimum inhibitory concentrations (MICs) against various fungal strains (including FLU-resistant clinical isolates of *C. albicans*). Hemolytic and cytotoxic activities assessed the toxic effects of these peptides on mammalian cells. The determination of fungicidal rates of these peptides was determined by time–kill kinetics. The fluorescent probe, flow cytometry, and electron microscopy were used to elucidate on their antifungal mechanisms.

## Materials and Methods

### Peptide Synthesis and Physiochemical Properties Analysis

Peptides were synthesized by Sangon Biotech (Shanghai, China) using N-9-fluorenylmethyloxycarbonyl (Fmoc) solid-phase synthesis and purified by reverse-phase high-performance liquid chromatography on Inertsil ODS-SP column (250 × 4.60 mm, with 5-μm internal particles) (GL Sciences, Tokyo, Japan). The fidelity and precise molecular mass were confirmed using electrospray ionization mass spectrometry (LCMS-2020, Shimadzu, Kyoto, Japan). Peptide purity used in the biologic assay was higher than 95%. The physicochemical properties of the peptides were calculated using the bioinformatics program HeliQuest^1^. The helical wheel projection was performed online using the helical wheel projection^2^. The secondary structure content of the peptide was estimated online by the K2D3 server^3^ (Louis-Jeune et al., 2012).

### Secondary Structural Analysis

Secondary structure analysis of the peptide was performed using a J-820 spectropolarimeter (Jasco, Tokyo, Japan) equipped with a 1-mm-path-length quartz cell at 25°C. The peptides were dissolved in 10-mM phosphate-buffered saline (PBS) (pH 7.4) or 30-mM sodium dodecyl sulfate (SDS) (Sigma-Aldrich, St. Louis, MO, United States) to give a final concentration of 150 μM. CD spectra were collected at a wavelength ranging from 190 to 250 nm with a scan rate of 100 nm/min, and each spectrum was the average of three scans. The following equation calculated the mean residue ellipticity (θ_*M*_, deg cm^2^ dmol^–1^):

$$\theta_{M}=\left(\theta_{o\,b\,s}\times 1,000\right)÷\left(c\times l\times n\right)$$

where θ_*obs*_ is the observed ellipticity (mdeg), c is the concentration (mM) of peptide solution, l is the path length (mm), and n is the number of peptide residues.

### Fungal Strains

*Candida albicans* cgmcc 2.2086, *Candida tropicalis* cgmcc 2.1975, and *Candida parapsilosis* cgmcc 2.3989 were purchased from the China General Microbiological Culture Collection Center (Beijing, China). Clinical isolated *C. albicans* sp3902, *C. albicans* sp3903, *C. albicans* sp3931, and *C. albicans* sp3876 were kindly provided by the School of Basic Medical Sciences, Nanchang University (Jiangxi, China). Clinical isolated *C. albicans* 56453, *C. albicans* 56214, *C. albicans* 14926, *C. albicans* 17546, and *C. albicans* 58288 were kindly provided by Zhongshan Hospital Affiliated to Fudan University (Shanghai, China).

### Antifungal Activity Assay

The antifungal activity of the peptide was determined using the broth microdilution method previously described (Yang et al., 2019). FLU (Sigma-Aldrich, Shanghai, China) and amphotericin B (AmB) (Sigma-Aldrich, St. Louis, MO, United States) were used as controls to compare the antifungal efficacies of the designed peptides.

Briefly, colonies from 24-h culture grown in yeast extract peptone dextrose (YPD) (AOBOXA, Beijing, China) agar medium were suspended in sterile saline to obtain stock inoculum suspensions with concentrations of optical density at 625 nm (OD625) of 0.08–0.1. The working suspensions were made by 1:1,000 dilutions of the stock suspensions with Roswell Park Memorial Institute (RPMI) 1640 (Gibco, Thermo Fisher Science, Inc.) buffered with morpholinepropanesulfonic acid (MOPS) (Sigma-Aldrich, St. Louis, MO, United States) at pH 7.0. The final concentrations of the working inoculum suspensions of the isolates were 1–2 × 10^3^ colony-forming units (CFU)/ml, as determined by quantitative colony counts on YPD agar medium. Peptides and antifungal agents were twofold serially diluted in 0.2 bovine serum albumin (Sigma-Aldrich, Shanghai, China) and RPMI-MOPS medium and then mixed with equal volumes of prepared fungal suspension in 96-well plates. After that, each well had a 100-μl mixture, and the final concentration ranges of FLU, AmB, and peptides were 256 to 0.5, 64 to 0.03, and 64 to 1 μM, respectively. The microdilution plates were incubated at 28°C for 48 h. The MICs were determined by the absorbance at 492 nm with a microplate reader as the lowest peptide concentration that inhibited 99% of the fungal growth. Fifty-microliter samples of each well were further removed and plated on YPD agar plates. The plates were incubated at 28°C for 48 h. The minimum fungicidal concentrations (MFCs) were determined as the lowest peptide concentration that completely killed fungal cells.

### Time–Kill Kinetics Assay

The killing kinetics of the peptides against *C. albicans* was recorded in terms of CFU per milliliter at different times of incubation. *C. albicans* cgmcc 2.2086 and *C. albicans* 56241 were diluted to approximately 10^5^ CFU/ml and incubated with peptides at concentrations of 1 × MIC and 2 × MIC. The mixtures were incubated at 37°C. At the predetermined time interval (10, 30, 60, and 120 min), the fungal suspensions were diluted with PBS and plated on YPD agar plates, and colonies were counted after 48 h of incubation. Results were obtained from three independent experiments.

### Hemolysis Assays

Hemolytic activities of the peptide were determined according to the method previously described (Lyu et al., 2019). The experimental protocol was reviewed and approved by the ethics committee of the Northeast Agriculture University Hospital, and the experimental method was carried out in accordance with the approved guidelines and regulations [NEAU-(2011)-9]. Fresh heparinized human whole blood from a healthy donor was centrifuged at 1,000 *g* for 5 min at 4°C. The obtained erythrocytes were washed three times and resuspended in PBS. Subsequently, serial dilutions of the peptides were mixed with erythrocytes and incubated for an hour at 37°C. Erythrocytes treated with PBS or 0.1% Triton X-100 (Sigma-Aldrich, Shanghai, China) were used as a negative or positive control, respectively. After centrifugation, the supernatant was transferred into a new 96-well plate, and released hemoglobin was measured using a multimode microplate reader (Infinite M200 Pro, Tecan, Switzerland) at 570 nm. The percent lysis was calculated according to the following equation:

$$hemolysis\left(\%\right)=\left[\left(A-A_{0}\right)÷\left(A_{t}-A_{0}\right)\right]$$

where *A* is the absorbance of the peptide sample, *A*_0_ and *A*_*t*_ represent 0 and 100% hemolysis determined in 10-mM PBS and 0.1% Triton X-100, respectively. Results were derived from three independent experiments, each performed in triplicate.

### Cytotoxicity Assays

The cytotoxic effects of the peptides on porcine intestinal epithelial cell line (IPEC-J2) and porcine mammary epithelial cells (PMEC) were determined using 3-(4,5-dimethylthiazol-2-yl)-2,5-diphenyltetrazolium bromide (MTT) assays. Viable cells contain NAD(P)H-dependent oxidoreductase enzymes, which reduce the MTT reagent to formazan, an insoluble crystalline product with a deep purple color. The darker the solution, the greater the number of viable, metabolically active cells. This method was performed as previously described (Chou et al., 2019). Briefly, cells were seeded in 96-well cell culture plates with approximately 5,000 cells per well. After overnight culture at 37°C in 5% carbon dioxide, the cells were treated with various concentrations of peptide for 24 h. Subsequently, cells were incubated with 50 μl of MTT (0.5 mg/ml) (Sigma-Aldrich, Shanghai, China) for 4 h. After incubation, 96-well plates were centrifuged at 1,000 *g* for 5 min, and the supernatants were discarded, and 150 μl of dimethyl sulfoxide (Solarbio, Beijing, China) was added to dissolve the formazan crystals. Finally, the absorbance at 570 nm was measured with a multimode microplate reader (Infinite M200 Pro, Tecan, Switzerland). Results were derived from three independent experiments, each performed in triplicate.

### Calculation of the Therapeutic Index

The therapeutic index (TI) is a quantitative measurement of the relative safety of the peptide. It is calculated by the ratio of HC10 (hemolytic activity) to GM (antimicrobial activity). HC10 is the minimal hemolytic concentration that induced a 10% hemolysis of human erythrocytes. GM is the geometric mean of the MIC values of a peptide against all the tested *C. albicans*. The larger the TI, the better the cell selectivity the peptide has.

### Drug Resistance Experiment

Drug resistance was induced by treating *C. albicans* repeatedly with antifungal agents, as previously described (Chou et al., 2019). Briefly, MIC testing was first conducted for tested peptides and antifungal agents, as described earlier. At the end of incubation (28°C for 48 h), the fungal cells growing in the well with the half-MIC concentration were harvested and adjusted to an OD625 of 0.08 to 0.1. After a 1:1,000 dilution with the RPMI-MOPS medium, the inoculum was subjected to the next passage MIC testing, and the process was repeated for 11 passages.

### Membrane Potential Assays

The alteration of membrane potential in peptide-treated *C. albicans* was detected using the membrane potential-sensitive fluorescent probe, 3,3′-dipropylthiadicarbocyanine iodide [DiSC3(5)] (Sigma-Aldrich, Shanghai, China), as previously described (Qi et al., 2010). In brief, *C. albicans* cells in mid-logarithmic growth-phase were harvested and suspended to an OD600 of 0.05 with 5-mM 4-(2-hydroxyethyl)-1-piperazineethanesulfonic acid buffer (pH 7.4, containing 20-mM glucose). After the addition of 0.4-μM DiSC3(5), samples were incubated for 90 min in the dark to obtain a stable reduction in fluorescence, and then, potassium chloride (4 M) was added to the cell suspension to give a final concentration of 100 mM. Subsequently, 2 ml of cell suspension was added to a 1-cm quartz cuvette and mixed with peptides at different concentrations. The changes in fluorescence were recorded for 900 s with an F-4500 fluorescence spectrophotometer (Hitachi, Japan) at excitation and emission wavelengths of 622 and 670 nm, respectively.

### Flow Cytometry Analysis

Analysis of the membrane integrity of *C. albicans* cgmcc 2.2086 after peptide treatment was performed by flow cytometry. As previously described (Li et al., 2016), *C. albicans* cells were harvested at the logarithmic phase and adjusted to a cell density of 1 × 10^6^ CFU/ml in RPMI-MOPS medium. Cell suspensions were incubated for 90 min with peptides at desired concentrations. Subsequently, treated cells were incubated with 10-μg/ml propidium iodide (PI) (Solarbio, Beijing, China) for 15 min, followed by washing and resuspension in PBS. PI fluorescence was collected by a FACScan instrument (Becton-Dickinson, San Jose, CA).

### Membrane Morphological Observation

Morphological alteration in *C. albicans* cells after peptide treatment was visualized by electron microscopy. For the scanning electron microscope (SEM) sample preparation, logarithmic *C. albicans* cgmcc 2.2086 were harvested and resuspended to an OD600 of 0.2 and incubated for 90 min with peptide at the desired concentration. After incubation, cells were collected through centrifugation and fixed overnight with 2.5% (v/v) glutaraldehyde at 4°C. After that, samples were dehydrated with an ascending ethanol series (50, 70, 90, and 100%). Dried samples were transferred to a mixture (1:1, v/v) of ethanol and tertiary butanol for 20 min, followed by pure tertiary butanol for 1 h. Specimens were dried and coated with gold and visualized using a HITACHI S-4800 SEM (Hitachi, Japan).

For transmission electron microscope (TEM) analysis, microbial samples were initially prepared as described earlier for SEM analysis. After prefixation with 2.5% glutaraldehyde overnight, cell pellets were washed three times with PBS and postfixed with 2% osmium tetroxide in PBS for 70 min. Samples were washed twice with PBS, followed by dehydration with a graded ethanol series (50, 70, 90, and 100%) and immersed in pure epoxy resin in a constant-temperature incubator overnight. Finally, specimens were sectioned using an ultramicrotome, stained with uranyl acetate and lead citrate, and observed using a HITACHI H-7650 TEM (Hitachi, Japan).

### Intracellular Reactive Oxygen Species Production

The level of intracellular reactive oxygen species (ROS) was measured by the fluorometric assay, as previously described (Wang et al., 2018). *C. albicans* cgmcc 2.2086 cells were harvested at the logarithmic phase and adjusted to a final concentration of OD600 of 0.6 and incubated with different concentrations of peptides for 60 min at 37°C. After treatment, fungal cells were stained with 10-μM 2′,7′-dichlorofluorescein diacetate (DCFH-DA) for 60 min. At the end of incubation, cells were washed twice with PBS to remove the excessive DCFH-DA. The fluorescence intensities were recorded (excitation 488 nm and emission 525 nm) with a multimode microplate reader (Infinite M200 Pro, Tecan, Switzerland). Results were derived from three independent experiments, each performed in triplicate.

### Statistical Analysis

All data were expressed as mean ± standard deviation (SD). Differences were analyzed using one-way ANOVA or a Student’s *t*-test. Statistical analyses were performed with SPSS software 20.0 (Chicago, IL, United States). A *p*-value of 0.05 or less was considered statistically significant. HC10 and IC50 values were calculated by using probit regression in SPSS 20.0.

## Results

### Peptide Characteristics

The sequences and key physicochemical properties of the peptides are summarized in Table 1. Accurate molecular masses of the synthesized peptides were consistent with their theoretical molecular masses, indicating the successful synthesis of all designed peptides. As shown in Table 1, peptides with 18 amino acid residues presented seven net charges. Hydrophobicity (H) values were calculated by the summation of hydrophobicity of all amino acids divided by the sequence length of the peptide. These values decreased in the order of KW3, RW3, KF3, RF3, KV3, and RV3, corresponding to reducing values.

**TABLE 1:** Amino acid sequences and key physicochemical parameters of the peptides.

**Peptides**	**Sequence**	**Theoretical M_*av*_**	**Measured M_*av*_^*a*^**	**Charge**	**H^*b*^**
KV	KIGKVL-NH_2_	655.87	655.89	+3	–^*c*^
KV2	KIGKVLLVKGIK-NH_2_	1,294.72	1,294.75	+5	0.457
KV3	KIGKVLLVKGIKKIGKVL-NH_2_	1,933.57	1,933.54	+7	0.457
RV3	RIGRVLLVRGIRRIGRVL-NH_2_	2,101.65	2,101.64	+7	0.450
KF3	KIGKFLLFKGIKKIGKFL-NH_2_	2,077.70	2,077.67	+7	0.552
KW3	KIGKWLLWKGIKKIGKWL-NH_2_	2,194.81	2,194.78	+7	0.628
RF3	RIGRFLLFRGIRRIGRFL-NH_2_	2,245.78	2,245.78	+7	0.545
RW3	RIGRWLLWRGIRRIGRWL-NH_2_	2,362.89	2,362.88	+7	0.622

*^*a*^Molecular average mass (M_*av*_) was measured by electrospray ionization mass spectrometry (ESI MS). ^*b*^Hydrophobicity (H) was calculated using the online program HeliQuest (http://heliquest.ipmc.cnrs.fr/cgi-bin/ComputParamsV2.py). ^*c*^Hydrophobicity (H) of KV was not available due to the shorter chain length than minimum chain length required for calculation.*

### Secondary Structures

**FIGURE 1:**
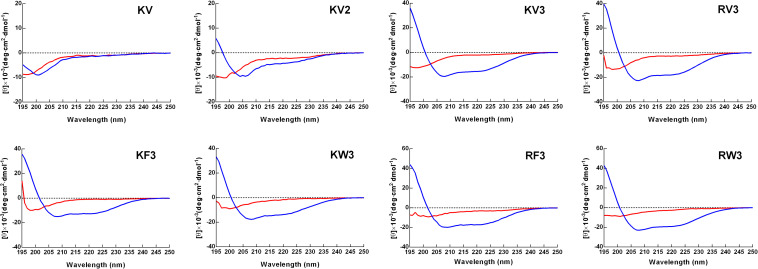
Circular dichroism (CD) spectra of designed peptides. Spectra were obtained at a peptide concentration of 150 μM in 10-mM phosphate-buffered saline (PBS) (pH 7.4) (red line) and 30-mM sodium dodecyl sulfate (SDS) (blue line).

Secondary structure transitions that were triggered by environmental alterations were investigated using CD spectroscopy. Mimicry of the microbial cell membrane environment was done using an anionic surfactant SDS. The designed peptides displayed unordered structures in an aqueous solution (10-mM PBS) and adopted α-helical structures (double minima at 208 and 222 nm) in the membrane mimicry environment (30-mM SDS). However, the KV still appeared as an unordered structure in 30-mM SDS (Figure 1). Accurate α-helical contents of the tested peptides were calculated from CD spectral data using online server K2D3, and the results are presented in Table 2. Compared with KV2, KV3 (which consisted of 18 amino-acid residues) showed a 42.52% increase in α-helical content in the secondary structure (Table 2). Valine-containing peptides KV3 and RV3, with 52.15 and 57.35% α-helix, respectively, exhibited a strong α-helical propensity, whereas peptides containing Phe (KF3 and RF3) exhibited low α-helix contents (39.89 and 43.33%, respectively). The incorporation of tryptophan changed the α-helix content to a lesser extent. KW3 and RW3 showed a 2.78% decrease and a 4.2% increase in helical contents compared with their valine-containing counterparts, KV3 and RV3, respectively.

**TABLE 2:** Percentage of α-helical structure in designed peptides in different solutions.

**Peptides**	**PBS^a^**		**SDS^b^**	
	**(θ) 222^c^**	**α-helix (%)^d^**	**(θ)222^c^**	**α -helix (%)^d^**
KV	−1,168.30	3.25	−1,449.66	5.10
KV2	−2,281.50	2.97	−4,138.67	9.63
KV3	−2,055.96	3.22	−15,067.66	52.15
RV3	−2,499.47	3.18	−17,452.07	57.35
KF3	−753.64	5.34	−12,478.98	39.89
KW3	−1,050.36	3.30	−13,046.52	49.34
RF3	−3,068.10	2.68	−17,014.84	43.33
RW3	−2,088.51	4.83	−18,453.47	61.55

*^*a*^Peptides were dissolved in 10-mM phosphate-buffered saline (PBS) (pH 7.4). ^*b*^Peptides were dissolved in 30-mM sodium dodecyl sulfate (SDS). ^*c*^Mean residue ellipticities (θ) (deg cm^2^ dmol^–1^) at wavelength 222 nm. ^*d*^α-Helical structure content was calculated online using K2D3 (http://cbdm-01.zdv.uni-mainz.de/andrade/k2d3/).*

### Antifungal Activities

The antifungal activities of the designed peptides are summarized in Table 3. The GMs of the MIC values of the peptides against *C. albicans* strains were calculated and presented in Table 4. KV and KV2 were inactive against all *Candida* strains, whereas KV3 exhibited a slight antifungal activity with MIC values ranging from 16 to 64 μM on *Candida* strains (Table 3). Substituting Lys residues in KV3 with Arg (designated as RV3) resulted in a twofold increase in anticandidal activities on *C. albicans*. Aromatic amino acid substitutions improved the antifungal activities of the peptides. KW3, RF3, and RW3, whose GM values were 5.28, 6.96, and 3.48 μM, respectively, exhibited the strongest antifungal activities against *C. albicans* (Table 4). Clinically isolated *C. albicans* strains that were resistant to FLU were highly susceptible to the three peptides. Melittin, an α-helix peptide, was used as a positive control to evaluate the antifungal effects of the α-helical peptides. Table 3 shows that melittin displayed strong inhibition effects on the growth of *C. albicans*. The prevalence of non-*C. albicans* strains have been increasing over time (Berkow and Lockhart, 2017). Therefore, the antifungal activities of these peptides against non-*C. albicans* strains were also evaluated. Table 3 shows that these peptides were also shown to be active against *C. tropicalis* and *C. parapsilosis*. The MFCs of the peptides were also determined, and the results were presented in Supplementary Table 1. The MFC values of peptides were approximately two times comparable with their MICs. Compared with the fungistatic agent FLU, the designed peptides were fungicidal.

**TABLE 3:** Antifungal activity of the peptides.

	**MIC (μM)^a^**										
	**KV**	**KV2**	**KV3**	**RV3**	**KF3**	**KW3**	**RF3**	**RW3**	**Melittin**	**FLU^b^**	**AmB^c^**
*C. albicans* cgmcc 2.2086	>64	>64	32	32	32	4	8	4	4	2	1
*C. albicans* 56452	>64	>64	32	16	16	4	8	4	8	>256	1
*C. albicans* 56214	>64	>64	32	16	16	4	4	2	4	>256	1
*C. albicans* 14936	>64	>64	>64	32	64	8	8	4	4	32	0.25
*C. albicans* 17546	>64	>64	64	64	64	8	8	8	8	>256	0.25
*C. albicans* 58288	>64	>64	64	32	32	8	8	4	4	8	0.5
*C. albicans* sp3902	>64	>64	64	32	16	2	8	2	2	16	0.25
*C. albicans* sp3903	>64	>64	64	32	32	4	8	2	4	16	0.13
*C. albicans* sp3931	>64	>64	64	32	32	8	8	4	8	1	0.25
*C. albicans* sp3876	>64	>64	16	32	64	8	4	4	4	>256	0.25
*C. tropicalis* cgmcc 2.1975	>64	>64	16	8	8	2	8	2	4	4	1
*C. parapsilosis* cgmc*c* 2.3989	>64	>64	32	16	32	8	16	4	2	4	2

*^*a*^Antifungal activity of the peptides was determined using minimum inhibitory concentration (MIC). MIC was representative consensus value of at least three independent experiments, each performed in triplicate. ^*b*^Fluconazole (FLU) is a conventional triazole antifungal agent. ^*c*^Amphotericin B (AmB) is a conventional polyene antifungal agent.*

**TABLE 4:** Specificity of the peptides.

**Peptides**	**GM (μM)^a^**	**HC10 (μM)^b^**	**TI^c^**
KV3	48.5	>64	2.64
RV3	29.86	>64	4.29
KF3	32	>64	4.00
KW3	5.28	2.69	0.51
RF3	6.96	>64	18.39
RW3	3.48	3.81	1.09
Melittin	4.59	0.91	0.20

*^*a*^GM, the geometric mean of the minimum inhibitory concentration (MIC) values of the peptides against tested *Candida albicans* strains. When no detectable antifungal activity against *C. albicans* was observed at 64 μM, a value of 128 μM was used for the calculation of GM. ^*b*^HC10, the hemolytic concentration of the peptides that induced 10% hemolysis of human blood red cells. ^*c*^Therapeutic index (TI) is calculated as HC10/GM. Higher TI values represent greater specificity of the peptides.*

### Hemolytic Activity and Cytotoxicity

**FIGURE 2:**
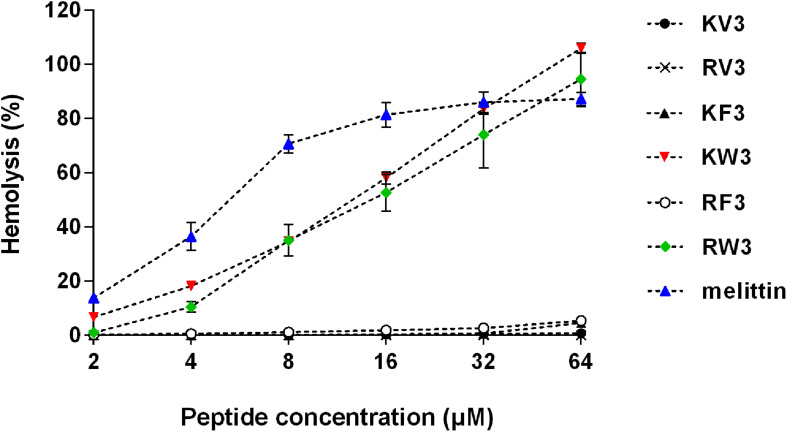
Hemolytic activity of the designed peptides. Human erythrocytes were incubated with peptides (2–64 μM) at 37°C for 1 h followed by the measurement of released hemoglobin at 570 nm. Values are means ± SD, *n* = 3.

The hemolytic activity of peptides was analyzed in human erythrocytes. Peptide concentrations that induced 10% hemolysis were calculated and presented in Table 4. Figure 2 shows that most of the peptides exhibited slight hemolytic activities at the highest tested concentration (64 μM). However, KW3 and RW3 exhibited strong hemolytic activities. KW3 and RW3 at a concentration of 2.69 and 3.81 μM, respectively, induced a 10% hemoglobin release (Table 4). With an HC10 value of 0.91 μM, melittin exhibited the highest hemolytic activity.

**FIGURE 3:**
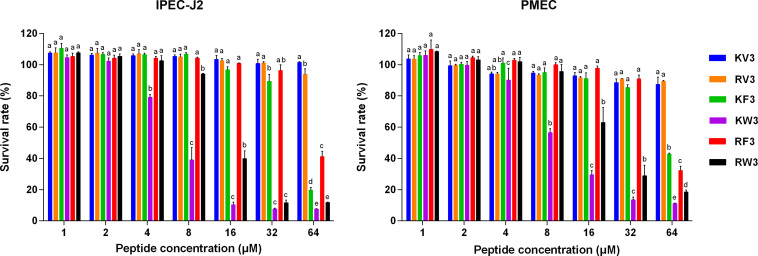
Cytotoxicity of the designed peptides on IPEC-J2 cells and PMEC cells. Cells were treated with peptides (1–64 μM) for 24 h, and cell viability was determined by 3-(4,5-dimethylthiozol-2-yl)-2,5-diphenyltetrazolium bromide (MTT) assay. Data were analyzed by one-way ANOVA with Tukey multiple comparison test. Values are means ± SD, *n* = 3. Different superscript letters (a–e) indicated significant mean differences, *p* < 0.05.

The cytotoxicity profiles of the peptides on IPEC-J2 and PMEC cells were determined using the MTT assay. Figure 3 shows that KV3 and RV3 had negligible cytotoxic effects on IPEC-J2 and PMEC cells. However, the antifungal activities of these peptides were not observed. At the respective concentrations of 4 and 8 μM, KW3 and RW3 started to exhibit statistically significant cytotoxic effects. KW3 (IC50 = 6.84 μM on IPEC-J2 cells and 10.26 μM on PMEC cells) and RW3 (IC50 = 14.78 μM on IPEC-J2 cells and 22.29 μM on PMEC cells) were the most toxic peptides (Figure 3 and Supplementary Table 2). KF3 and RF3 maintained high cell survival rates at 32 μM, but a sharp rise in cytotoxicity on both cell lines was observed at 64 μM.

### Cell Selectivity and Time–Kill Kinetics

The TI is a parameter representing the cell selectivity of a peptide. It is calculated by the ratio of HC10 (hemolytic activity) to GM (antimicrobial activity). As shown in Table 4, RF3 displayed strong cell selectivity properties toward *C. albicans* over human erythrocytes. Its TI value (18.39) was 20 times higher than that of melittin. In contrast, the other two effective peptides, KW3 and RW3, exhibited poor cell selectivities due to their high hemolytic activities.

**FIGURE 4:**
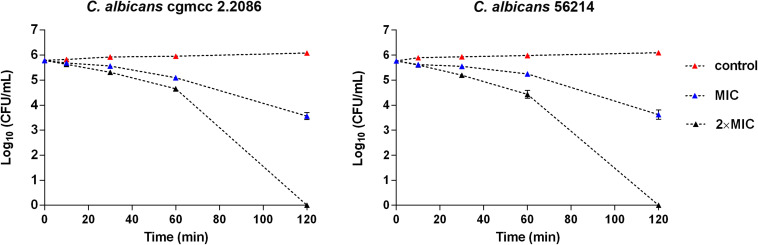
Time–killing kinetics of RF3 against *C. albicans* cgmcc 2.2086 and *C. albicans* 56241. Fungal suspensions were incubated with peptides at concentrations of 1 × MIC and 2 × MIC. At various time intervals, samples were diluted and plated for colony counts. Values are means ± SD, *n* = 3.

Time–kill kinetics of RF3 was performed on *C. albicans* cgmcc 2.2086 and *C. albicans* 56214 at concentrations of 1 × MIC and 2 × MIC, respectively. As shown in Figure 4, RF3 displayed concentration-dependent killing. After 2-h treatment, the lower concentration (1 × MIC) of RF3 did not eliminate the colonies but produced 2.2-log CFU and 2.1-log CFU reductions in *C. albicans* cgmcc 2.2086 and *C. albicans* 56214 strains, respectively. At 2 × MIC concentration, no fungal growth was observed after incubation.

### Development of Resistance

**FIGURE 5:**
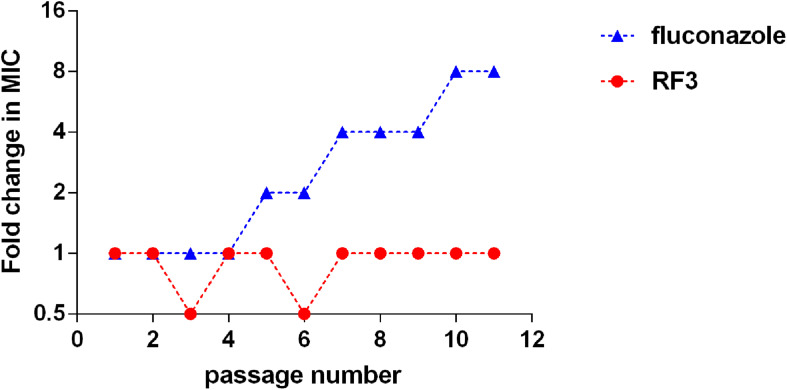
Serial passage of *C. albicans* cgmcc 2.2086 in the presence of sub-MIC concentration of RF3 or fluconazole.

To investigate the potential of fungi to develop drug resistance against RF3, *C. albicans* cgmcc 2.2086 were exposed toward sub-MIC concentrations of RF3 over 10 passages. Conventional antifungal agent FLU was used as control. As shown in Figure 5, exposure to a subinhibitory concentration of FLU induced an eightfold MIC-increase of FLU in *C. albicans* following 10 passages, whereas the MIC of RF3 remained constant during 10 passages. This result suggested that fungal resistance to RF3 was not generated easily.

### Cytoplasmic Membrane Depolarization

**FIGURE 6:**
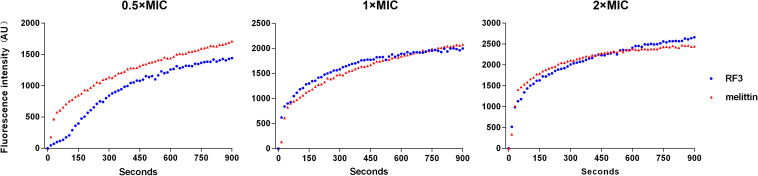
Peptide-triggered cytoplasmic membrane depolarization of *C. albicans* cgmcc 2.2806. Membrane potential variations induced by different concentrations (0.5 × MIC, 1 × MIC, and 2 × MIC) of RF3 and melittin were monitored using voltage-sensitive dye 3,3′-dipropylthiadicarbocyanine (DiSC3–5). Fluorescence intensity was monitored at an excitation wavelength of 622 nm and an emission wavelength of 670 nm as a function of time.

The ability of RF3 to depolarize the cytoplasmic membrane of *C. albicans* was evaluated by the potentiometric probe DiSC3(5). DiSC3(5) accumulates on hyperpolarized membranes and translocates into the lipid bilayer, leading to fluorescence self-quenching. Upon depolarization, the dye is rapidly released into the medium resulting in increased fluorescence (Te Winkel et al., 2016). The RF3 and melittin that induced cytoplasmic membrane depolarization of *C. albicans* cgmcc 2.2086 were monitored for a period of 900 s. As shown in Figure 6, RF3 depolarized fungal cytoplasmic membranes in a concentration-dependent manner. However, at a concentration of 0.5 × MIC, melittin elicited stronger membrane depolarization. RF3 was more effective in depolarizing the cytoplasmic membrane of *C. albicans* cgmcc 2.2086 at 1 × MIC and 2 × MIC concentrations.

### Membrane Permeabilization

**FIGURE 7:**
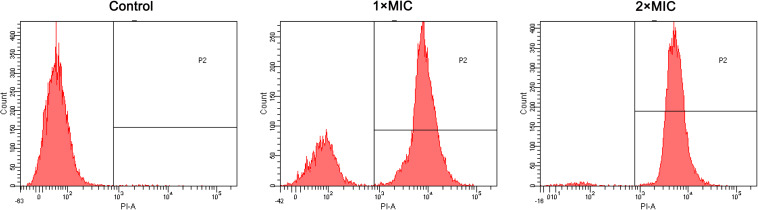
RF3 induced membrane permeabilization of *C. albicans* cgmcc 2.2086. Fluorescence intensity of propidium iodide (PI) (10 μg ml^–1^) after 90-min treatment with different concentrations (1 × MIC and 2 × MIC) of RF3 was detected by flow cytometry.

The effect of RF3 on membrane permeability was determined by flow cytometry using PI, a membrane-impermeable fluorescent dye. Nucleic acids stained with PI suggested a loss of membrane integrity (Lam et al., 2016). As shown in Figure 7, RF3 played a strong role in destroying the permeability of *C. albicans* cgmcc 2.2086 cell membrane. This activity was dependent on the concentration of the peptide. In the absence of RF3, the percentage of PI-positive cells was 0.4. After treatment with RF3 at concentrations of 1 × MIC and 2 × MIC, the cells were found to be more damaged, with 73 and 97.7% PI fluorescence signals, respectively.

### Morphological Observation

**FIGURE 8:**
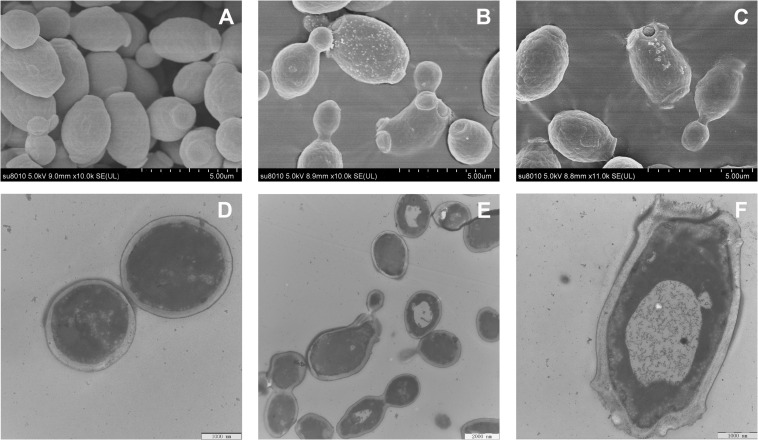
Morphological changes in RF3-treated *C. albicans* cgmcc 2.2086. Scanning electron microscope (SEM) micrographs of *C. albicans*: **(A)** control, without peptide; **(B,C)** RF3 at 1 × MIC. Transmission electron microscope (TEM) micrographs of *C. albicans*: **(D)** control, without peptide; **(E,F)** RF3 at 1 × MIC.

The effect of RF3 treatment on *C. albicans* cell morphology was visualized using SEM and TEM. Figure 8 shows that untreated cells had intact and smooth membranes. However, the membrane surfaces of RF3-treated cells (Figures 8B,C) appeared rough, shrunken, and irregular. Numerous bleb-like structures were also observed on their surfaces. Membrane integrity and ultrastructural alterations were investigated using TEM. Compared with the untreated cells (Figure 8D), treatment with RF3 caused significant cell membrane breaks and fractures. Also, RF3 treatment-induced large cytoplasmic vacuoles, as shown in Figures 8E,F.

### Intracellular Reactive Oxygen Species Production

**FIGURE 9:**
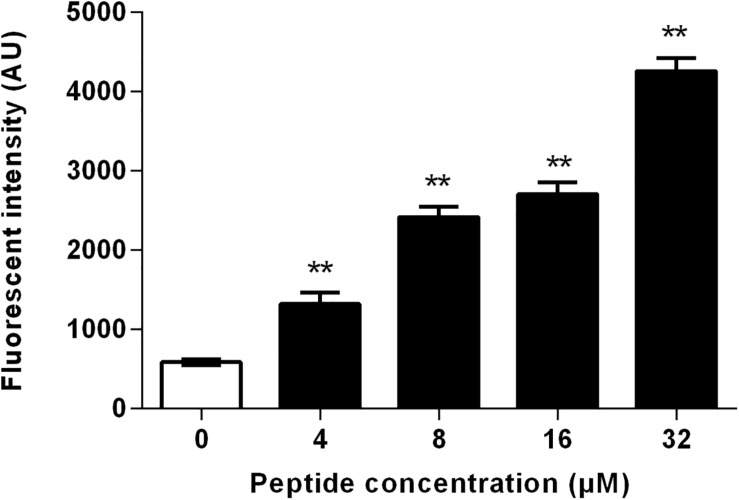
Effect of RF3 on intracellular reactive oxygen species (ROS) formation of *C. albicans* cgmcc 2.2086. Cells were treated with RF3 (4–32 μM) for 60 min, and the level of ROS was measured using 2′,7′-dichlorofluorescein diacetate (DCFH-DA). **p < 0.01 (two-tailed Student’s *t*-test) compared with control. Values are means ± SD, *n* = 3.

The level of intracellular ROS was measured using DCFH-DA, which is oxidized by ROS to generate fluorescent 2′,7′-dichlorodihydrofluorescein (Wang et al., 2015). As shown in Figure 9, the ROS generation increased markedly in the presence of RF3. Treatment with RF3 resulted in a concentration-dependent increase in intracellular ROS level. Compared with the control group, treatment with 1 × MIC RF3 induced a 4.1-fold increase in fluorescence intensity.

## Discussion

The defective immune system is one of the leading causes of the rise of fungal infection incidences in the past decade. Fungal infections due to *Candida* species are the most common in this group. *C. albicans* have developed resistance to clinically available antifungal agents (Wu et al., 2017). The membrane disruption antifungal mechanism of AMPs offers a great avenue for overcoming the drug resistance problem (Wang et al., 2019).

Studies have shown that an increase in peptide length results in increased antimicrobial activity within a certain range (Chou et al., 2016; Wu et al., 2017). Hence, we adopted a signal and double addition of KV to obtain KV2 and KV3. Amphiphilicity is an essential, but, controversial parameter that affects the antimicrobial activity of AMPs. It is a factor that is believed to be crucial in the antimicrobial activity of AMPs. Studies have shown that imperfect amphiphilic peptides had better antimicrobial activities compared with the corresponding perfect amphiphilic peptides (Wiradharma et al., 2013; Wang et al., 2018). Due to reverse tandem duplication, KV3 had an imperfect but relatively better amphiphilicity (Supplementary Figure 1). Additionally, the amino acid composition that determines the cationic and hydrophobic properties and conformation are associated with the antimicrobial and cell-selective properties of the AMPs (Lum et al., 2015; Wu et al., 2015). It has been suggested that Arg-containing peptides have better bactericidal efficacies than Lys-containing peptides. Aromatic amino acids with bulky side chains serve as anchors in the interactions between AMPs and microbial membranes (Marsh et al., 2007; Zhu et al., 2015). The arginine-substituted analog (designated as RV3) of KV3 was first designed by replacing the Lys residues with Arg. Valine residues in KV3 and RV3 were then replaced by aromatic hydrophobic amino acids, Trp and Phe, respectively, and KF3, KW3, RF3, and RW3 were obtained.

Peptides display unordered conformations in aqueous environments and convert to well-defined structures upon associating with microbial membranes. These conformational transitions influence the insertion of AMPs into the cell membrane (Ciociola et al., 2016; Guilhelmelli et al., 2016). Except for KV, which was too short of forming stable secondary structures, the designed peptides folded into α-helical structures in 30-mM SDS (Figure 1). Compared with KV, increased helical propensities were observed in KV2 and KV3 (Figure 1 and Table 2). The hydrogen-bonding interactions along the helical backbone that result in α-helical structures can be enhanced by increasing the chain length (Wiradharma et al., 2011). In comparison with Val-containing peptides, KV3 and RV3 peptides with Phe as the hydrophobic residue (KF3 and RF3) had lower helical contents (39.89 and 43.33%) (Table 2). This observation was in agreement with a previous finding that Phe residues are less conducive for helical formation. Differences in helical contents between peptides containing different hydrophobic amino acids arise from the intrinsic helix-forming properties of the amino acids. The propensity of Val toward the helical structure is larger than that of Phe and Trp (Blaber et al., 1993). However, Trp residues did not have that much influence on the helical content. One possible explanation is that the structure of a peptide is not only determined by the intrinsic properties of a single residue; the contact between amino acid residues are also determinants of the final conformation.

Increasing the chain length resulted in enhanced α-helical structures and increased net charges. The net amount is the most important characteristic of AMPs. The first step in antimicrobial actions is the electrostatic interactions between peptides and microbial membranes. The fungal cell wall is composed of glucan–chitin complexes and mannoproteins. Most of the mannoproteins carry N-linked glycans that have outer chains consisting of α-linked mannose units. Phosphorylation of the mannosyl side chains contribute to the negative charges of fungal walls (Lipke and Ovalle, 1998). Large fractions of the negatively charged phosphatidylinositol are also found in fungi (van der Weerden et al., 2013). Higher cationic charges enhance electrostatic interactions between peptides and negatively charged fungal membranes. The propensity toward helical structures causes a great membrane binding affinity. Enhancement of the properties, as mentioned earlier, improve the antimicrobial activities of AMPs. In this study, the 12 amino acid residue peptides, KV2 (with five net charges), was ineffective against all fungal strains, whereas the 18 amino acid residue peptides, KV3 (with seven net charges), exhibited slightly high antifungal activities (Table 3). The antifungal activities of these peptides were also improved by altering their amino acid compositions. Substituting Lys with Arg residues increased the antifungal activity of KV3 (Table 3). The guanidinium group of Arg has more dispersed positive charges than the single amine of Lys. This property enhances peptide-membrane electrostatic interactions that result in improved antimicrobial activities (Liu et al., 2007; Yang et al., 2019). Aromatic residues play an essential role in peptide membrane anchoring abilities (Marsh et al., 2007). Aromatic amino acid replacement altered the hydrophobicity of peptides. Hydrophobicity is important in antimicrobial activities of the peptides, as it facilitates peptide–membrane interactions and governs the extent of AMP insertions into the cell membrane (Chen et al., 2007). In the present study, peptides containing aromatic amino acid residues exhibited higher hydrophobicity than the Val-containing peptides. Tryptophan-containing peptides (KW3 and RW3) exhibited the highest hydrophobicity (Table 1). This phenomenon was due to the high intrinsic hydrophobicity of Trp than that of Phe (Damodaran, 2008). Antifungal activities of aromatic amino acid-containing peptides were enhanced by improving their hydrophobic properties. KW3, RF3, and RW3, respectively, displayed 9-, 7-, and 14-fold antifungal activities on *Candida* sp. compared with the parent peptide, KV3 (Table 4). Interestingly, RV3 and KV3 did not exhibit good antifungal activities, although they possessed a strong α-helical signature (Tables 2, 3). The importance of hydrophobicity in the interactions of RV3 and KV3 with fungal membranes was, therefore, greater than the helical structural conformation.

Toxicity tests of new therapeutic compounds are essential in drug development. As shown in Figures 2, 3, the toxic effects of these peptides increased with increasing hydrophobicity. Increased hydrophobicity allows the depth of insertion of the peptides into microbial membranes, and deep penetration into the hydrophobic interiors of mammalian cells is enhanced by increased hydrophobicity. This increased antimicrobial activities and toxicity (Jiang et al., 2008). At a concentration of 64 μM, the cytotoxic effects of RF3 were observed, and cell selectivity properties of RF3 were lost (Figure 3). The therapeutic indices were calculated by determining the ratio of hemolytic activity to the antifungal activity. RF3 displayed strong cell selectivity properties toward *C. albicans* over human erythrocytes, as indicated by the highest TI value of 18.93 (Table 4). RF3 was screened and its fungicidal mechanisms determined.

Most α-helical antifungal peptides exert antifungal activities by interfering with membrane permeability. The presence of negatively charged phosphatidylinositol in fungi makes the cytoplasmic membrane highly electronegative. This leads to the promotion in the strength of electrostatic interactions between the membrane and cation-bearing peptides. We report that RF3 depolarized cytoplasmic membranes in a concentration-dependent manner (Figure 6). This result indicates that the dissipation of membrane potential might be involved in channel or pore formation, thereby allowing the passage of ions or large molecules and thus leading to cytoplasmic membrane dysfunction and cell death (Te Winkel et al., 2016). The PI uptake assay also supported the changes in membrane permeability and integrity induced by RF3. PI, a membrane-impermeable dye, was used to detect cells that had permeable membranes. PI enters into cells and binds to nucleic acids and fluoresces red (Choi and Lee, 2014). RF3 treatment significantly increased membrane permeability (Figure 7). Fractured cell membranes and vesicular cytoplasm were observed after RF3 treatment (Figure 8). These results confirmed that RF3 exerted antifungal activities by disrupting membrane integrity. Compared with other kinds of novel antifungal agents that are designed base on the structure of FLU (Fakhim et al., 2017), this non-specific physical membrane-disrupting mechanism of RF3 reduced the development of antifungal resistance. Studies indicate that ROS production is involved in antifungal mechanisms of certain peptides. For example, the human salivary peptide histatin 5 has been shown to inhibit *C. albicans* by inducing the formation of ROS (Helmerhorst et al., 2001). Also, the fungicidal activity of protonectin, an AMP isolated from the venom of the neotropical social wasp *Agelaia pallipes pallipes*, is associated with the accumulation of ROS (Wang et al., 2015). Levels of ROS influence the proliferation and differentiation of fungal organisms (Belozerskaya and Gessler, 2007). However, excessive ROS production due to environmental stress causes hyperoxidation leading to the loss of cell functions and cell death (Gessler et al., 2007). Intracellular ROS generation was significantly increased by RF3 treatment (Figure 9).

## Conclusion

In conclusion, peptides with better hydrophobic properties exhibited greater antifungal activities. However, enhanced hydrophobicity also led to higher toxicity. Optimization of hydrophobicity is essential for cell selectivity. Among the designed peptides, RF3 exhibited the best cell selectivity and antifungal activity, specifically against FLU-resistant clinical *C. albicans* isolates. The dual-targeted antifungal mechanism of RF3 reported earlier reduced the probability of resistant development in fungi. These findings provide new insights into designs of antifungal peptides as alternative treatment options. Clearly, *in vivo* studies are required to elucidate the efficacy of RF3 in treating fungal infections fully. The mode of administration and pharmacokinetics also need further studies.

## Data Availability Statement

The raw data supporting the conclusions of this article will be made available by the authors, without undue reservation, to any qualified researcher.

## Ethics Statement

The experimental protocol was reviewed and approved by the Ethics Committee of the Northeast Agriculture University Hospital, and the experimental method was carried out in accordance with the approved guidelines and regulations. The participant provided her written informed consent to participate in this study.

## Author Contributions

YY and AS designed and conceived the experiments. YY, CW, and NG conducted the main experiments assay. LZ and YL analyzed the data. YY wrote the main manuscript text. SZ, JW, and AS supervised the work and revised the final version of the article. All authors contributed to the article and approved the submitted version.

## Conflict of Interest

The authors declare that the research was conducted in the absence of any commercial or financial relationships that could be construed as a potential conflict of interest.

## Footnotes

1 http://heliquest.ipmc.cnrs.fr/cgi-bin/ComputParamsV2.py

2 http://rzlab.ucr.Edu/scripts/wheel/wheel.cgi

3 http://cbdm-01.zdv.uni-mainz.de/~andrade/k2d3/

## References

[B1] Andes D. R. Safdar N. Baddley J. W. Alexander B. Brumble L. Freifeld A. (2016). The epidemiology and outcomes of invasive *Candida* infections among organ transplant recipients in the United States: results of the Transplant-Associated Infection Surveillance Network (TRANSNET). *Transpl. Infect. Dis.* 18 921–931. 10.1111/tid.12613 27643395

[B2] Belozerskaya T. A. Gessler N. N. (2007). Reactive oxygen species and the strategy of antioxidant defense in fungi: a review. *Appl. Biochem. Microbiol.* 43 506–515. 10.1134/S0003683807050031 18038677

[B3] Berkow E. L. Lockhart S. R. (2017). Fluconazole resistance in *Candida* species: a current perspective. *Infect. Drug Resist.* 10 237–245. 10.2147/IDR.S118892 28814889 PMC5546770

[B4] Bhetariya P. J. Sharma N. Singh P. Tripathi P. Upadhyay S. K. Gautam P. (2017). “Human fungal pathogens and drug resistance against azole drugs,” in *The Drug Resistance in Bacteria, Fungi, Malaria, and Cancer*, eds Arora G. Sajid A. Kalia V. (Cham: Springer), 387–428. 10.1007/978-3-319-48683-3_18

[B5] Blaber M. Zhang X. J. Matthews B. W. (1993). Structural basis of amino acid α helix propensity. *Science* 260 1637–1640. 10.1126/science.8503008 8503008

[B6] Broekaert W. F. Terras F. Cammue B. Osborn R. W. (1995). Plant defensins: novel antimicrobial peptides as components of the host defense system. *Plant Physiol.* 108 1353–1358. 10.1104/pp.108.4.1353 7659744 PMC157512

[B7] Chen Y. Guarnieri M. T. Vasil A. I. Vasil M. L. Mant C. T. Hodges R. S. (2007). Role of peptide hydrophobicity in the mechanism of action of α-helical antimicrobial peptides. *Antimicrob. Agents Chemother.* 51 1398–1406. 17158938 10.1128/AAC.00925-06 PMC1855469

[B8] Choi H. Lee D. G. (2014). Antifungal activity and pore-forming mechanism of astacidin 1 against *Candida albicans*. *Biochimie* 105 58–63. 10.1016/j.biochi.2014.06.014 24955933

[B9] Chou S. Shao C. Wang J. Shan A. Xu L. Dong N. (2016). Short, multiple-stranded β-hairpin peptides have antimicrobial potency with high selectivity and salt resistance. *Acta Biomater.* 30 78–93. 10.1016/j.actbio.2015.11.002 26546414

[B10] Chou S. Wang J. Shang L. Akhtar M. U. Wang Z. Shi B. (2019). Short, symmetric-helical peptides have narrow-spectrum activity with low resistance potential and high selectivity. *Biomater. Sci.* 7 2394–2409. 10.1039/C9BM00044E 30919848

[B11] Ciociola T. Giovati L. Conti S. Magliani W. Santinoli C. Polonelli L. (2016). Natural and synthetic peptides with antifungal activity. *Future Med. Chem.* 8 1413–1433. 10.4155/fmc-2016-0035 27502155

[B12] Damodaran S. (2008). *Amino Acids, Peptides and Proteins.* Boca Raton, FL: CRC Press.

[B13] Dou X. Gao N. Lan J. Han J. Yang Y. Shan A. (2019). TLR2/EGFR are two sensors for pBD3 and pEP2C induction by sodium butyrate independent of HDAC inhibition. *J. Agric. Food Chem.* 68 512–522. 10.1021/acs.jafc.9b06569 31870150

[B14] Fakhim H. Emami S. Vaezi A. Hashemi S. M. Faeli L. Diba K. (2017). In vitro activities of novel azole compounds ATTAF-1 and ATTAF-2 against fluconazole-susceptible and-resistant isolates of *Candida* species. *Antimicrob. Agents Chemother.* 61:e01106-16. 10.1128/AAC.01106-16 27795371 PMC5192144

[B15] Fesharaki S. H. Haghani I. Mousavi B. Kargar M. L. Boroumand M. Anvari M. S. (2013). Endocarditis due to a co-infection of *Candida albicans* and *Candida tropicalis* in a drug abuser. *J. Med. Microbiol.* 62 1763–1767. 10.1099/jmm.0.060954-0 23973985

[B16] Fisher M. C. Hawkins N. J. Sanglard D. Gurr S. J. (2018). Worldwide emergence of resistance to antifungal drugs challenges human health and food security. *Science* 360 739–742. 10.1126/science.aap7999 29773744

[B17] Ganz T. Lehrer R. I. (1998). Antimicrobial peptides of vertebrates. *Curr. Opin. Immunol.* 10 41–44. 9523109 10.1016/s0952-7915(98)80029-0

[B18] Gessler N. N. Aver’yanov A. A. Belozerskaya T. A. (2007). Reactive oxygen species in regulation of fungal development. *Biochemistry* 72 1091–1109. 10.1134/S0006297907100070 18021067

[B19] Guilhelmelli F. Vilela N. Smidt K. S. de Oliveira M. A. da Cunha Morales (2016). Activity of scorpion venom-derived antifungal peptides against planktonic cells of *Candida* spp. and *Cryptococcus neoformans* and *Candida albicans* biofilms. *Front. Microbiol.* 7:1844. 10.3389/fmicb.2016.01844 27917162 PMC5114273

[B20] Harioudh M. K. Sahai R. Mitra K. Ghosh J. K. (2017). A short non-cytotoxic antimicrobial peptide designed from Aβ29-40 adopts nanostructure and shows in vivo anti-endotoxin activity. *Chem. Commun.* 53 13079–13082. 10.1039/C7CC07547B 29168511

[B21] Helmerhorst E. J. Troxler R. F. Oppenheim F. G. (2001). The human salivary peptide histatin 5 exerts its antifungal activity through the formation of reactive oxygen species. *Proc. Natl. Acad. Sci. U.S.A.* 98 14637–14642. 10.1073/pnas.141366998 11717389 PMC64734

[B22] Jiang Z. Kullberg B. J. Van Der Lee H. Vasil A. I. Hale J. D. Mant C. T. (2008). Effects of hydrophobicity on the antifungal activity of α-helical antimicrobial peptides. *Chem. Biol. Drug Des.* 72 483–495. 10.1111/j.1747-0285.2008.00728.x 19090916 PMC2730115

[B23] Kodedová M. Sychrová H. (2017). Synthetic antimicrobial peptides of the halictines family disturb the membrane integrity of *Candida* cells. *Biochim. Biophys. Acta Biomembr.* 1859 1851–1858. 10.1016/j.bbamem.2017.06.005 28600071

[B24] Koehbach J. Craik D. J. (2019). The vast structural diversity of antimicrobial peptides. *Trends Pharmacol. Sci.* 40 517–528. 10.1016/j.tips.2019.04.012 31230616

[B25] Lacerda A. F. Pelegrini P. B. de Oliveira D. M. Vasconcelos É. A. Grossi-de-Sá M. F. (2016). Anti-parasitic peptides from arthropods and their application in drug therapy. *Front. Microbiol.* 7:91. 10.3389/fmicb.2016.00091 26903970 PMC4742531

[B26] Lam S. J. O’Brien-Simpson N. M. Pantarat N. Sulistio A. Wong E. H. Chen Y.-Y. (2016). Combating multidrug-resistant Gram-negative bacteria with structurally nanoengineered antimicrobial peptide polymers. *Nat. Microbiol.* 1:16162. 10.1038/nmicrobiol.2016.162 27617798

[B27] Le C.-F. Fang C.-M. Sekaran S. D. (2017). Intracellular targeting mechanisms by antimicrobial peptides. *Antimicrob. Agents Chemother.* 61:e02340-16. 10.1128/AAC.02340-16 PMC5365711 28167546

[B28] Lécorché P. Walrant A. Burlina F. Dutot L. Sagan S. Mallet J.-M. (2012). Cellular uptake and biophysical properties of galactose and/or tryptophan containing cell-penetrating peptides. *Biochim. Biophys. Acta Biomembr.* 1818 448–457. 10.1016/j.bbamem.2011.12.003 22182801

[B29] Li J. Koh J.-J. Liu S. Lakshminarayanan R. Verma C. S. Beuerman R. W. (2017). Membrane active antimicrobial peptides: translating mechanistic insights to design. *Front. Neurosci.* 11:73. 10.3389/fnins.2017.00073 28261050 PMC5306396

[B30] Li L. Sun J. Xia S. Tian X. Cheserek M. J. Le G. (2016). Mechanism of antifungal activity of antimicrobial peptide APP, a cell-penetrating peptide derivative, against *Candida albicans*: intracellular DNA binding and cell cycle arrest. *Appl. Microbiol. Biotechnol.* 100 3245–3253. 10.1007/s00253-015-7265-y 26743655

[B31] Limper A. H. Adenis A. Le T. Harrison T. S. (2017). Fungal infections in HIV/AIDS. *Lancet Infect. Dis.* 17 e334–e343. 28774701 10.1016/S1473-3099(17)30303-1

[B32] Lipke P. N. Ovalle R. (1998). Cell wall architecture in yeast: new structure and new challenges. *J. Bacteriol.* 180 3735–3740. 10.1128/JB.180.15.3735-3740.1998 9683465 PMC107352

[B33] Liu Z. Brady A. Young A. Rasimick B. Chen K. Zhou C. (2007). Length effects in antimicrobial peptides of the (RW) n series. *Antimicrob. Agents Chemother.* 51 597–603. 17145799 10.1128/AAC.00828-06 PMC1797765

[B34] Louis-Jeune C. Andrade-Navarro M. A. Perez-Iratxeta C. (2012). Prediction of protein secondary structure from circular dichroism using theoretically derived spectra. *Proteins* 80 374–381. 10.1002/prot.23188 22095872

[B35] Lum K. Y. Tay S. T. Le C. F. Lee V. S. Sabri N. H. Velayuthan R. D. (2015). Activity of novel synthetic peptides against *Candida albicans*. *Sci. Rep.* 5:9657. 10.1038/srep09657 25965506 PMC4603303

[B36] Lupetti A. Paulusma-Annema A. Senesi S. Campa M. van Dissel J. T. Nibbering P. H. (2002). Internal thiols and reactive oxygen species in candidacidal activity exerted by an N-terminal peptide of human lactoferrin. *Antimicrob. Agents Chemother.* 46 1634–1639. 10.1128/AAC.46.6.1634-1639.2002 12019068 PMC127236

[B37] Lv Y. Wang J. Gao H. Wang Z. Dong N. Ma Q. (2014). Antimicrobial properties and membrane-active mechanism of a potential α-helical antimicrobial derived from cathelicidin PMAP-36. *PLoS One* 9:e86364. 10.1371/journal.pone.0086364 24466055 PMC3897731

[B38] Lyu Y. Chen T. Shang L. Yang Y. Li Z. Zhu J. (2019). Design of Trp-rich dodecapeptides with broad-spectrum antimicrobial potency and membrane-disruptive mechanism. *J. Med. Chem.* 62 6941–6957. 10.1021/acs.jmedchem.9b00288 31276398

[B39] Marsh D. Jost M. Peggion C. Toniolo C. (2007). Lipid chain-length dependence for incorporation of alamethicin in membranes: electron paramagnetic resonance studies on TOAC-spin labeled analogs. *Biophys. J.* 92 4002–4011. 10.1529/biophysj.107.104026 17351010 PMC1868974

[B40] Methatham T. Boonchuen P. Jaree P. Tassanakajon A. Somboonwiwat K. (2017). Antiviral action of the antimicrobial peptide ALFPm3 from *Penaeus monodon* against white spot syndrome virus. *Dev. Comp. Immunol.* 69 23–32. 10.1016/j.dci.2016.11.023 27919648

[B41] Nett J. E. Andes D. R. (2016). Antifungal agents: spectrum of activity, pharmacology, and clinical indications. *Infect. Dis. Clin.* 30 51–83. 10.1016/j.idc.2015.10.012 26739608

[B42] Nguyen L. T. Haney E. F. Vogel H. J. (2011). The expanding scope of antimicrobial peptide structures and their modes of action. *Trends Biotechnol.* 29 464–472. 10.1016/j.tibtech.2011.05.001 21680034

[B43] Odds F. C. Brown A. J. Gow N. A. (2003). Antifungal agents: mechanisms of action. *Trends Microbiol.* 11 272–279. 12823944 10.1016/s0966-842x(03)00117-3

[B44] Pappas P. G. Rex J. H. Sobel J. D. Filler S. G. Dismukes W. E. Walsh T. J. (2004). Guidelines for treatment of candidiasis. *Clin. Infect. Dis.* 38 161–189. 10.1086/380796 14699449

[B45] Qi X. Zhou C. Li P. Xu W. Cao Y. Ling H. (2010). Novel short antibacterial and antifungal peptides with low cytotoxicity: efficacy and action mechanisms. *Biochem. Biophys. Res. Commun.* 398 594–600. 10.1016/j.bbrc.2010.06.131 20603106

[B46] Sanglard D. (2016). Emerging threats in antifungal-resistant fungal pathogens. *Front. Med.* 3:11. 10.3389/fmed.2016.00011 27014694 PMC4791369

[B47] Shao C. Tian H. Wang T. Wang Z. Chou S. Shan A. (2018). Central β-turn increases the cell selectivity of imperfectly amphipathic α-helical peptides. *Acta Biomater.* 69 243–255. 10.1016/j.actbio.2018.01.009 29355714

[B48] Te Winkel J. D. Gray D. A. Seistrup K. H. Hamoen L. W. Strahl H. (2016). Analysis of antimicrobial-triggered membrane depolarization using voltage sensitive dyes. *Front. Cell. Dev. Biol.* 4:29. 10.3389/fcell.2016.00029 27148531 PMC4829611

[B49] van der Weerden N. L. Bleackley M. R. Anderson M. A. (2013). Properties and mechanisms of action of naturally occurring antifungal peptides. *Cell. Mol. Life Sci.* 70 3545–3570. 23381653 10.1007/s00018-013-1260-1 PMC11114075

[B50] Vazquez J. A. (2017). Combination antifungal therapy: the new frontier. *Future Microbiol.* 2 115–139. 10.2217/17460913.2.2.115 17661650

[B51] Vos W. M. Kuipers O. P. Meer J. R. Siezen R. J. (1995). Maturation pathway of nisin and other lantibiotics: post-translationally modified antimicrobial peptides exported by Gram-positive bacteria. *Mol. Microbiol.* 17 427–437. 10.1111/j.1365-2958.1995.mmi_17030427.x 8559062

[B52] Wang J. Chou S. Yang Z. Yang Y. Wang Z. Song J. (2018). Combating drug-resistant fungi with novel imperfectly amphipathic palindromic peptides. *J. Med. Chem.* 61 3889–3907. 10.1021/acs.jmedchem.7b01729 29648811

[B53] Wang J. Dou X. Song J. Lyu Y. Zhu X. Xu L. (2019). Antimicrobial peptides: promising alternatives in the post feeding antibiotic era. *Med. Res. Rev.* 39 831–859. 10.1002/med.21542 30353555

[B54] Wang K. Dang W. Xie J. Zhu R. Sun M. Jia F. (2015). Antimicrobial peptide protonectin disturbs the membrane integrity and induces ROS production in yeast cells. *Biochim. Biophys. Acta Biomembr.* 1848 2365–2373. 10.1016/j.bbamem.2015.07.008 26209560

[B55] Whaley S. G. Berkow E. L. Rybak J. M. Nishimoto A. T. Barker K. S. Rogers P. D. (2016). Azole antifungal resistance in *Candida albicans* and emerging non-*albicans Candida* species. *Front. Microbiol.* 7:2173. 10.3389/fmicb.2016.02173 28127295 PMC5226953

[B56] Williams H. Campbell L. Crompton R. A. Singh G. McHugh B. J. Davidson D. J. (2018). Microbial host interactions and impaired wound healing in mice and humans: defining a role for BD14 and NOD2. *J. Invest. Dermatol.* 138 2264–2274. 10.1016/j.jid.2018.04.014 29723492

[B57] Wiradharma N. Khoe U. Hauser C. A. Seow S. V. Zhang S. Yang Y.-Y. (2011). Synthetic cationic amphiphilic α-helical peptides as antimicrobial agents. *Biomaterials* 32 2204–2212. 10.1016/j.biomaterials.2010.11.054 21168911

[B58] Wiradharma N. Sng M. Y. Khan M. Ong Z. Y. Yang Y. Y. (2013). Rationally designed α-helical broad-spectrum antimicrobial peptides with idealized facial amphiphilicity. *Macromol. Rapid Commun.* 34 74–80. 10.1002/marc.201200534 23112127

[B59] Wu H. Liu S. Wiradharma N. Ong Z. Y. Li Y. Yang Y. Y. (2017). Short synthetic α-helical-forming peptide amphiphiles for fungal keratitis treatment in vivo. *Adv. Healthc. Mater.* 6:1600777. 10.1002/adhm.201600777 28081296

[B60] Wu H. Ong Z. Y. Liu S. Li Y. Wiradharma N. Yang Y. Y. (2015). Synthetic β-sheet forming peptide amphiphiles for treatment of fungal keratitis. *Biomaterials* 43 44–49. 10.1016/j.biomaterials.2014.11.052 25591960

[B61] Yang Y. Wu D. Wang C. Shan A. Bi C. Li Y. (2020). Hybridization with insect cecropin A (1–8) improve the stability and selectivity of naturally occurring peptides. *Int. J. Mol. Sci.* 21:1470. 10.3390/ijms21041470 32098142 PMC7073140

[B62] Yang Z. He S. Wang J. Yang Y. Zhang L. Li Y. (2019). Rational design of short peptide variants by using Kunitzin-RE, an amphibian-derived bioactivity peptide, for acquired potent broad-spectrum antimicrobial and improved therapeutic potential of commensalism coinfection of pathogens. *J. Med. Chem.* 62 4586–4605. 10.1021/acs.jmed-chem.9b00149 30958004

[B63] Zhang L. Tong Y. Dai H. Cui J. Ren B. (2015). Synergistic combinations of antifungals and antivirulence agents to fight against *Candida albicans*. *Virulence* 6 362–371. 10.1080/21505594.2015.1039885 26048362 PMC4601232

[B64] Zhu X. Zhang L. Wang J. Ma Z. Xu W. Li J. (2015). Characterization of antimicrobial activity and mechanisms of low amphipathic peptides with different α-helical propensity. *Acta Biomater.* 18 155–167. 10.1016/j.actbio.2015.02.023 25735802

