# Supplementary material for: A Novel Dual-Targeted α-Helical Peptide With Potent Antifungal Activity Against Fluconazole-Resistant Candida albicans Clinical Isolates
Source: Front Microbiol. 2020 Sep 30;11:548620. doi: 10.3389/fmicb.2020.548620 (PMC7554340; doi:10.3389/fmicb.2020.548620)
Supplement: Supplementary file 1 [file Data_Sheet_1.docx]

Supplementary Material
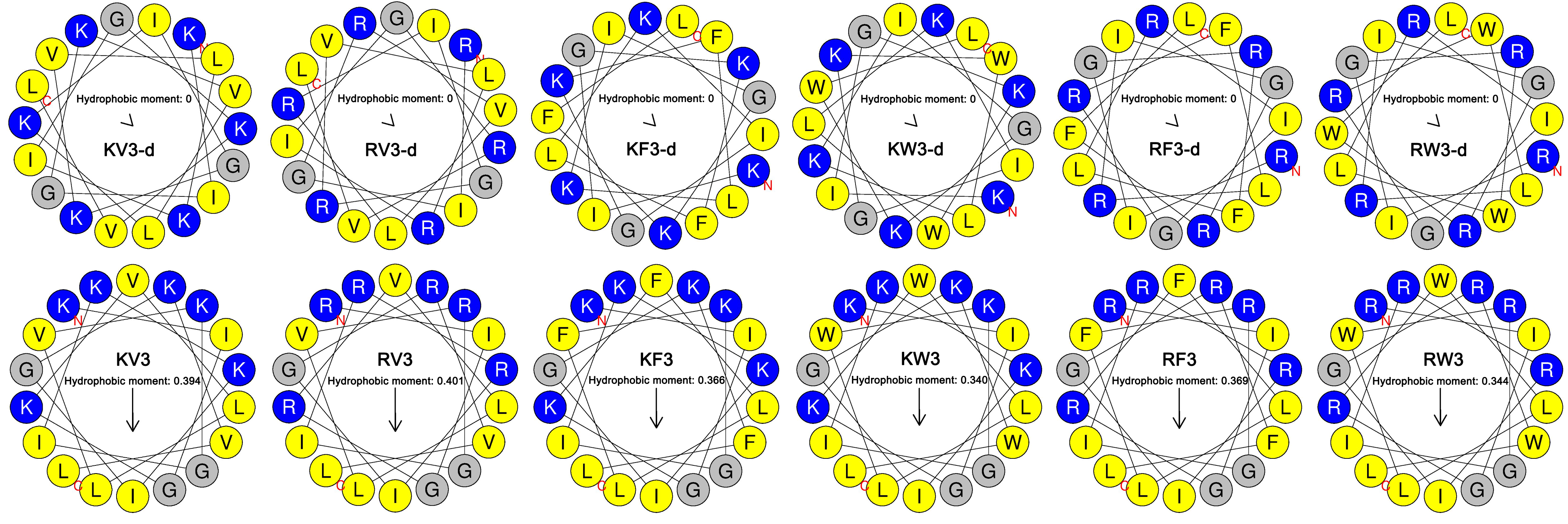


Supplementary Figure 1. Helical wheel diagrams of the peptides using direct duplication as the method to increase the length (top row) and peptides using reverse tandem duplication as the mothed to increase the length (bottom row). Among these helical wheel diagrams, by default the output presents the charged residues as blue, hydrophobicity residues as yellow and glycine as gray, respectively. The longer the arrow length, the greater the relative hydrophobic moments in the figure.

Supplementary Table 1 Minimum Fungicidal Concentrations of the Designed Peptides

|  | MFC^a^ (μM) | | | | | | | | | | |
| --- | --- | --- | --- | --- | --- | --- | --- | --- | --- | --- | --- |
|  | KV | KV2 | KV3 | RV3 | KF3 | KW3 | RF3 | RW3 | melittin | FLU^b^ | AmB^c^ |
| *C. albicans* cgmcc 2.2086 | >64 | >64 | 32 | 32 | 32 | 8 | 8 | 4 | 8 | 4 | 1 |
| *C. albicans* 56452 | >64 | >64 | 64 | 16 | 16 | 4 | 8 | 4 | 8 | >256 | 2 |
| *C. albicans* 56214 | >64 | >64 | 32 | 32 | 16 | 8 | 8 | 2 | 4 | >256 | 2 |
| *C. albicans* 14936 | >64 | >64 | >64 | 32 | 64 | 8 | 8 | 4 | 8 | >256 | 0.5 |
| *C. albicans* 17546 | >64 | >64 | 64 | 64 | 64 | 8 | 16 | 8 | 8 | >256 | 1 |
| *C. albicans* 58288 | >64 | >64 | 64 | 32 | 32 | 8 | 8 | 8 | 4 | >256 | 1 |
| *C. albicans* sp3902 | >64 | >64 | 64 | 32 | 16 | 2 | 8 | 2 | 2 | >256 | 1 |
| *C. albicans* sp3903 | >64 | >64 | 64 | 32 | 32 | 8 | 8 | 8 | 4 | 64 | 1 |
| *C. albicans* sp3931 | >64 | >64 | 64 | 32 | 32 | 8 | 8 | 4 | 8 | 64 | 1 |
| *C. albicans* sp3876 | >64 | >64 | 32 | 32 | 64 | 8 | 4 | 4 | 4 | >256 | 0.5 |
| *C.tropicalis* cgmcc 2.1975 | >64 | >64 | 16 | 16 | 8 | 2 | 8 | 2 | 8 | 32 | 2 |
| *C. parapsilosis* cgmc*c* 2.3989 | >64 | >64 | 32 | 32 | 32 | 8 | 16 | 4 | 2 | 8 | 2 |

^a^Minimum fungicidal concentrations (MFC) were determined as the lowest peptide concentration that completely killed fungal cells. MFC was representative consensus value of at least three independent experiments. ^b^Fluconzaole (FLU) is a conventional triazole antifungal agent. ^c^Amphotericin B(AmB) is a conventional polyene antifungal agent.

Supplementary Table 2 Cytotoxicity of peptides on IPEC-J2 and PMEC cell lines

| peptides | IC50^a^ | |
| --- | --- | --- |
|  | IPEC-J2 | PMEC |
| KV3 | >64 | >64 |
| RV3 | >64 | >64 |
| KF3 | 48.47 | 59.97 |
| KW3 | 6.84 | 10.26 |
| RF3 | 59.75 | 53.99 |
| RW3 | 14.78 | 22.29 |

^a^50% inhibitory concentration (IC50) of the designed peptides against IPEC-J2 and PMEC cell lines.
